# Straw Mulching and Nitrogen Fertilization Affect Diazotroph Communities in Wheat Rhizosphere

**DOI:** 10.3389/fmicb.2021.658668

**Published:** 2021-05-21

**Authors:** Songhe Chen, Xiaoling Xiang, Hongliang Ma, Petri Penttinen, Jiarong Zhao, Han Li, Rencai Gao, Ting Zheng, Gaoqiong Fan

**Affiliations:** ^1^Key Laboratory of Crop Eco-Physiology and farming system in Southwest China, Ministry of Agriculture, College of Agronomy, Sichuan Agricultural University, Chengdu, China; ^2^Department of Microbiology College of Resources, Sichuan Agricultural University, Chengdu, China

**Keywords:** straw mulching, nitrogen fertilization, diazotroph community, *nifH* gene, abundance

## Abstract

Diazotrophs that carry out the biological fixation of atmospheric dinitrogen (N_2_) replenish biologically available nitrogen (N) in soil and are influenced by the input of inorganic and organic substrates. To date, little is known about the effects of combined organic substrate addition and N fertilization on the diazotroph community composition and structure in purple soils. We investigated the effects of N fertilization and straw mulching on diazotroph communities by quantifying and sequencing the *nifH* gene in wheat rhizosphere. The abundance and richness of diazotrophs were greater the higher the fertilization level in the mulched treatments, whereas in the nonmulched treatments (NSMs), richness was lowest with the highest N fertilization level. The abundance and α-diversity of diazotrophs correlated with most of the soil properties but not with pH. At the genus level, the relative abundances of *Azospirillum*, *Bacillus*, and *Geobacter* were higher in the NSMs and those of *Pseudacidovorax*, *Skermanella*, *Azospira*, *Paraburkholderia*, *Azotobacter*, *Desulfovibrio*, *Klebsiella*, and *Pelomonas* in the mulched treatments. The differences in community composition between the mulched and the NSMs were associated with differences in soil temperature and soil organic carbon and available potassium contents and C:N ratio. Overall, straw mulching and N fertilization were associated with changes in diazotroph community composition and higher abundance of *nifH* gene in alkaline purple soils.

## Introduction

Nitrogen (N) is an essential element that plays a crucial role in agroecosystems (Feng et al., 2018). N transformation processes in the N cycle, e.g., N fixation (dinitrogen fixation), nitrification, and organic N mineralization, provide inorganic N to fulfill the requirements of plants (Luo et al., 2018; Pereg et al., 2018). Microbes play an important role in the soil N cycle by regulating N availability and transformation (Canfield et al., 2010). N_2_-fixing bacteria and archaea, diazotrophs, convert atmospheric N to bioavailable N (Levy-Booth et al., 2014; Chen et al., 2021). Diazotrophs may promote the growth of cereals, even though this has been mostly attributed to their other plant growth-promoting abilities instead of N fixation (Rosenblueth et al., 2018). Diazotrophs are highly diverse and include members of several bacterial phyla, e.g., Proteobacteria, Firmicutes and Cyanobacteria, and Archaea (Rösch et al., 2002). The *nifH* gene in the diazotrophs encodes a subunit of the nitrogenase enzyme and is highly conserved across the bacterial and archaeal domains, making *nifH* a suitable molecular biomarker to detect diazotrophic microbes (Zehr et al., 2003; Collavino et al., 2014).

Agricultural management practices, e.g., mulching and fertilization, affect the physiochemical properties of soil (Wang Y.S. et al., 2017). Diazotrophs are sensitive to soil physiochemical properties, including soil pH (Wang Y.S. et al., 2017; Fan et al., 2018), soil organic carbon (SOC) content (Collavino et al., 2014; Yang et al., 2019), C and N availability (Wang C. et al., 2017; Hu et al., 2019; Yang et al., 2021), phosphorus content (Tang et al., 2017), soil moisture (Penton et al., 2016), and C:N ratio (Wang C. et al., 2017; Liu et al., 2019). However, the effects of soil physiochemical properties on the diazotrophs have been variable. For example, the effects of soil pH on the diversity and abundance of soil diazotrophs were inconsistent in different soil types (Wang Y.S. et al., 2017; Fan et al., 2018; Lin et al., 2018). Likewise, N availability has been found to have both stimulatory (Perez et al., 2014; Reardon et al., 2014) and inhibitory effects (Zhalnina et al., 2015; Wang C. et al., 2017) on the diversity of diazotrophs.

The effects of fertilization on diazotroph communities are a growing concern (Meng et al., 2012; Wang C. et al., 2017). Long-term experiments have shown that the application of N fertilizers has a great impact on the diazotroph community composition (Meng et al., 2012; Reardon et al., 2014; Fan et al., 2019). For example, long-term synthetic fertilizer application can decrease the diversity and change the community structure of diazotrophs (Wang C. et al., 2017). However, in several studies, N fertilization did not affect the abundance and community composition of *nifH*-containing microbes in agricultural soils (Wakelin et al., 2007; Mårtensson et al., 2009; Sun et al., 2015). In general, the addition of organic substrates, e.g., by straw mulching, into soil provides a source of energy and nutrients to support the growth of diazotrophs (Tang et al., 2012; Liao et al., 2018). To date, little is known about the effects of combined straw mulching and N fertilization on the diazotroph community composition in purple soils. Thus, it is important to explore the effects of long-term N application alone and in combination with organic substrates, e.g., straw, on the abundance, diversity, and community composition of diazotrophs to develop sustainable cultivation practices.

The nutrient-rich, neutral or alkaline purple soils in the Sichuan Basin of southwestern China have a complex mineral composition and are characterized with high fertility and suitability to various crops (Wang et al., 2015). However, nitrification activity is high in purple soils, which has resulted in severe soil erosion and N loss *via* nitrate leaching and runoff (Zhou et al., 2012; Zhao et al., 2018; Zhong et al., 2019). Therefore, increasing N availability is essential for the sustainability of agriculture in the purple soil regions (Wang et al., 2015). This could be at least partially achieved through biological N fixation by diazotrophs.

For this study, we established a field experiment to monitor the effects of inorganic N fertilizer and straw mulching on the community composition of diazotrophs in purple soil. We applied quantitative PCR (qPCR) and high-throughput sequencing targeting the *nifH* gene to assess variation in the diazotroph community. Our objectives were to (1) evaluate the effects of N fertilization and straw mulching on the abundance, diversity, and community structure of diazotrophs; (2) identify the diazotroph genera affected by N fertilization and straw mulching; and (3) determine the relationships between dominant diazotroph genera and soil properties. We hypothesized that fertilization and straw incorporation would change soil properties, in particular the C and N levels, that would further affect the diazotroph communities; higher C to N ratios were expected to be associated with higher abundance and α-diversity of diazotrophs.

## Materials and Methods

### Experimental Site and Sampling

The straw mulch no-tillage experiment was established in 2015 at Renshou experimental base (30°04′N, 104°13′E) of the Agricultural College of Sichuan Agricultural University, China. The station is located in an area with a humid subtropical monsoon climate, an annual average temperature of 17.4°C and a mean annual precipitation of 1,009 mm. The soil is a typical alkaline purple soil. The basic physicochemical properties of the 0–20-cm soil layer in 2015 were as follows: pH 7.82 (soil:water = 1:2.5), organic carbon content 9.78 g kg^–1^, total nitrogen (TN) content 0.83 g kg^–1^, total phosphorus (TP) content 0.86 g kg^–1^, and total potassium content 13.96 g kg^–1^.

The split plot design experiment had straw mulching as the main factor and nitrogen level as the subplot factor with four 30 m^2^ (6 m × 5 m) replicate plots per treatment. The straw mulching rates were 0 (NSM: no straw mulching) and 8,000 (SM: straw mulching) kg ha^–1^. N fertilizer levels were 0 (N0), 120 (N1), and 180 (N2) kg ha^–1^. P and K fertilizers were applied to all the plots as 75 kg P_2_O_5_ ha^–1^ and 75 kg K_2_O ha^–1^, respectively.

Soil samples were collected on March 20, 2019, at the wheat anthesis stage. Fifteen wheat plants per plot were collected gently using a spade, and the soil attached to the roots was pooled into one composite rhizosphere soil sample per plot. The samples were transported on ice in a constant-temperature box to the laboratory and stored at −80°C.

### Soil Physicochemical Analysis

Soil pH and SOC, TN, TP, available P (AP), and available potassium (AK) contents were analyzed as described previously (Liu et al., 2020). NH_4_^+^-N and NO_3_^–^-N were extracted from fresh soil with 2 mol L^–1^ KCl and determined using colorimetry (P7 Double Beam UV–Visible Spectrophotometer, Mapada Inc. Shanghai, China) (Liu et al., 2020). Soil temperature was measured using a soil moisture and water potential instrument (Em50G, Decagon Devices Inc. Pullman, United States).

### DNA Extraction and Quantification of *nifH* Gene Abundance

DNA was extracted from 0.5 g of soil using the Fast DNA Spin Kit for Soil (MP Biomedicals, CA, United States) according to the manufacturer’s instructions. The purity and concentration of the extracted DNA were detected by using 2% agarose gel electrophoresis and a Nanodrop 2000 spectrophotometer (Thermo Scientific, United States). The extracted DNA was stored at −20°C.

The *nifH* gene copy number was determined using real-time qPCR on an ABI7500 Fast Real-Time PCR System (Applied Biosystems Inc. United States). The *nifH* fragments were amplified in triplicate using primers *PolF* (5′-TGCGAYCCSAARGCBGACTC-3′) and *PolR* (5′-ATSGCCATCATYTCRCCGGA-3′) (Poly et al., 2001). Amplification was done in a 20-μl reaction including 10 μl SYBR Green Master Mix (Applied Biosystems, United States), 0.25 μl (10 μM) of each primer, 1 μl (1–10 ng) DNA template or 1 μl sterilized distilled water in the negative control, and 8.5 μl double-distilled water (ddH_2_O). Amplification was initiated by denaturation at 95°C for 3 min, followed by 35 cycles of denaturation at 95°C for 10 s, annealing at 55°C for 30 s, extension at 72°C for 30 s, and reading the plate at 80°C (Lin et al., 2018).

The standards for qPCR were made by cloning a *nifH* gene fragment from mixed soil DNA samples. The *nifH* fragment was amplified as described above using pmD^®^18-T Vector (TaKaRa, Dalian, China) according to the manufacturer’s instructions. Based on a translated nucleotide query (Altschul et al., 1997), the fragment was 88% similar with *nifH* from *Ruminococcus* sp. (sequence ID MBE6845760.1). Plasmid DNA was extracted using a Plasmid Miniprep Kit (Biomiga, Santiago, United States), and the plasmid concentration was measured with a spectrophotometer (Nanodrop 2000, Thermo Scientific, Wilmington, United States) (Liao et al., 2018). As the sequences of the vector and PCR inserts were known, the copy number of *nifH* was calculated directly from the concentration of extracted nonlinearized plasmid DNA. A standard curve was done using 10-fold serial dilutions from 1.13 × 10^3^ to 1.13 × 10^9^ *nifH* copies (Wang et al., 2012). Since Nanodrop measurements are prone to overestimate DNA concentration (Kapp et al., 2015), the plasmid copy numbers were likely overestimated. However, this overestimation was not expected to affect comparisons between treatments. The amplification efficiency was 81.8% with *R*^2^-value of 0.999 and slope of −3.85. No amplification was detected in the negative controls. The detected CT values in samples were all within the range of the standard curve.

### *nifH* Gene Sequencing and Bioinformatics Analysis

The *nifH* amplicons of approximately 360 bp were amplified using the primers PolF with a unique sample identifying barcode and PolR. Amplification was done in triplicate 25 μl reactions including 1 μl of each 10 μM primer, 2 μl (20 ng) of template DNA or 2 μl sterilized distilled water in the negative control, and 12.5 μl of MasterMix containing Taq DNA polymerase, PCR buffer, Mg^2+^ and dNTPs (CWBIO, China), and 8.5 μl double distilled water (ddH_2_O). The amplification program included initial denaturation at 95°C for 3 min, followed by 40 cycles of 95°C for 30 s, 60°C for 60 s, and 72°C for 60 s, and a final extension at 72°C for 10 min (Gaby et al., 2018). The triplicate PCR products of each sample were mixed and purified with a gel extraction kit (TIANGEN, Inc., Beijing, China) according to the manufacturer’s instructions and quantified using a Qubit 2.0 fluorimeter (Invitrogen, Carlsbad, CA, United States). No amplification was detected in the negative controls. Purified amplicons were pooled in equimolar concentrations and sequenced using MiSeq Reagent Kit V3 for paired-end sequencing on an Illumina MiSeq platform at Shanghai Personal Biotechnology Co., Ltd (Shanghai, China).

### Bioinformatics Analysis

The raw sequences were demultiplexed and low-quality sequences were removed using the QIIME2 pipeline (Bolyen et al., 2019). Reads with an average quality score below 20 were filtered out and the remaining reads were trimmed at 200 and 450 bp as minimum and maximum length, respectively. Chimeric sequences were removed using UCHIME against a reference alignment (Edgar et al., 2011). A total of 1,965,677 high-quality sequences were obtained from 24 samples. The UPARSE pipeline was used to cluster sequences into operational taxonomic units (OTUs) (Edgar, 2013). Sequences with 97% similarity were assigned to an OTU (Edgar, 2010), after removing the singleton OTUs, generating 5,283 OTUs.

### Data Analysis

The statistical analyses were conducted using SPSS 22.0 software (International Business Machine, Armonk, New York, United States). Differences in soil properties, *nifH* abundance, and α-diversity were tested using two-way ANOVA followed by Fisher’s least significant difference (LSD) test. The *nifH* abundance data were log_10_ transformed and checked for normality prior to the test. The relationships between soil properties, *nifH* abundance, and α-diversity were estimated using Pearson correlation analysis. β-Diversity was visualized using weighted UniFrac distance-based principal coordinate analysis (PCoA) in R package “ape” (Hu et al., 2020). Hierarchical cluster analysis was performed using the “hclust” function with the average linkage algorithm in R version 3.5.1 (R Development Core Team, 2012). Corrplot in R was used to visualize the results of Pearson correlation analysis. Differences in diazotroph community structures were tested using permutational multivariate analysis of variance (PERMANOVA) in the R package vegan (Oksanen et al., 2012). The relationships between community variation and environmental factors were tested using redundancy analysis (RDA) with 999 permutations (Legendre and Anderson, 1999). Linear discriminant analysis effect size (LEfSe) analysis with default parameters was used to detect differentially abundant taxa (Segata et al., 2011).

## Results

### The Abundance and Diversity of the Diazotrophic Community

**FIGURE 1:**
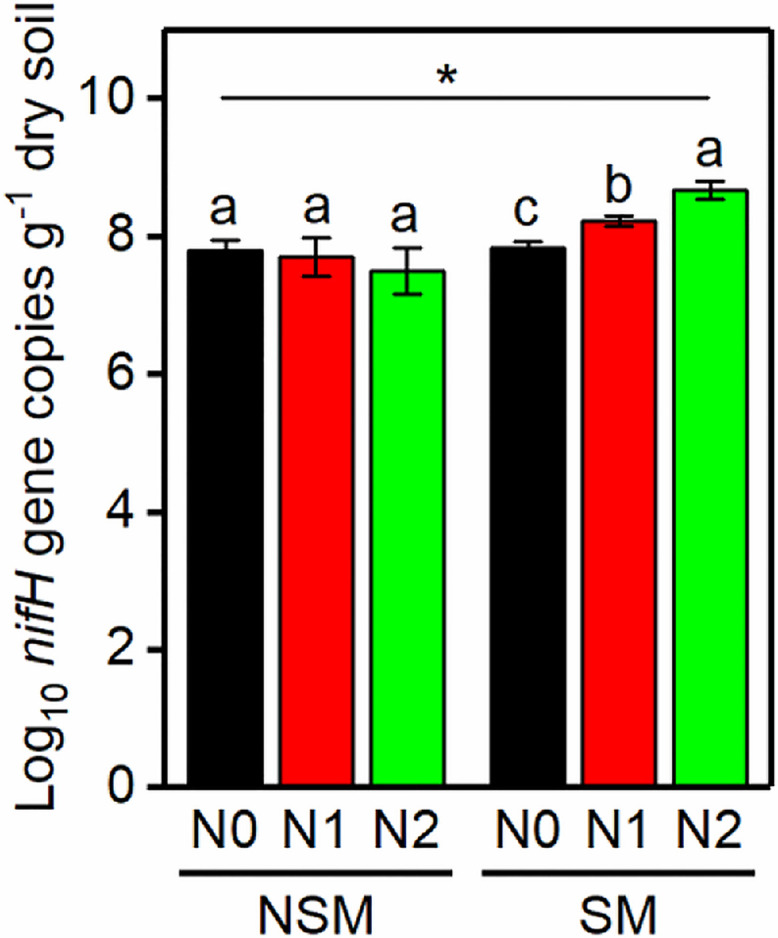
The abundance of *nifH* gene copies in wheat rhizosphere soil under straw mulching and nitrogen fertilization treatments. NSM, no straw mulching; SM, straw mulching; N0, no nitrogen; N1, 120 kg N ha^− 1^; N2, 180 kg N ha^− 1^. Data shown as mean ± SD. *indicates statistically significant difference (*P* < 0.05) between NSM and SM. Different letters above columns indicate statistically significant difference (*P* < 0.05) between N fertilizer levels within NSM and SM.

**FIGURE 2:**
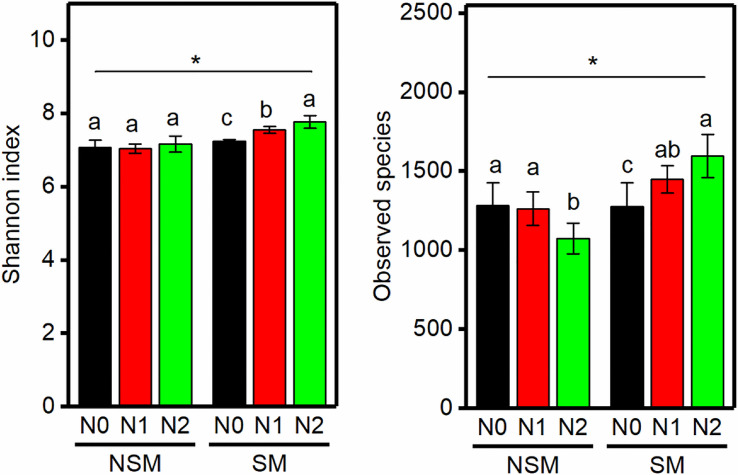
The α-diversity of the diazotrophs in wheat rhizosphere soil under straw mulching and nitrogen fertilization treatments. NSM, no straw mulching; SM, straw mulching; N0, no nitrogen; N1, 120 kg N ha^− 1^; N2, 180 kg N ha^− 1^. Data shown as mean ± SD. *indicates statistically significant difference (*P* < 0.05) between NSM and SM. Different letters above columns indicate statistically significant difference (*P* < 0.05) between N fertilizer levels within NSM and SM.

The *nifH* gene copy numbers ranged from 3.2 × 10^7^ to 4.7 × 10^8^ per g soil, with the lowest and the highest numbers in the NSMN2 and SMN2 treatment, respectively (Figure 1). In the mulched treatment (SM), the abundance of *nifH* gene was higher, the higher the nitrogen fertilization level (*P* < 0.05) (Figure 1). Compared with the nonmulched treatment (NSM), the diversity and richness of diazotrophs were higher in the SM (*P* < 0.05) (Figure 2). Furthermore, N fertilization resulted in higher diazotroph diversity and richness in the SM. In the NSM, richness was lowest in the highest fertilization level (*P* < 0.05) (Figure 2).

### The Composition of the Diazotroph Community

**FIGURE 3:**
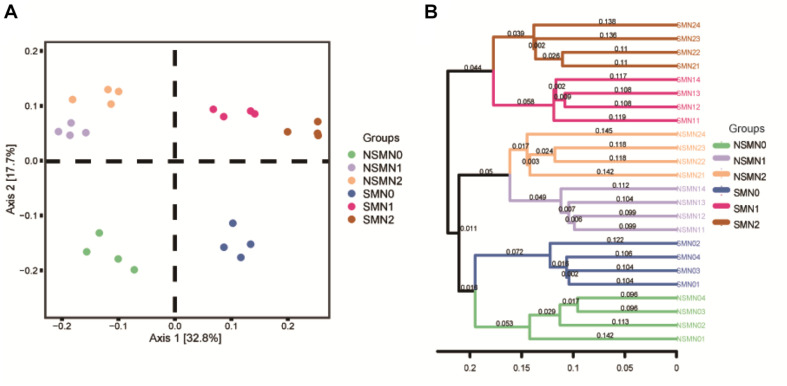
Principal coordinates analysis **(A)** and hierarchical clustering **(B)** of diazotroph communities in wheat rhizosphere soil under straw mulching and nitrogen fertilization treatments. NSM, no straw mulching; SM, straw mulching; N0, no nitrogen; N1, 120 kg N ha^− 1^; N2, 180 kg N ha^− 1^.

The *nifH* communities were distinct in all the six mulching–fertilizer level combinations (*P* < 0.05) (Supplementary Table 1). The first and the second principal coordinates explained 50.5% of the variation in the community composition in the PCoA (Figure 3A). The mulched and NSMs were clearly separated along axis 1, and the nonfertilized treatments NSM0 and SMN0 were separated from the fertilized treatments (*P* < 0.05) (Figure 3A). Similarly, the samples were divided into three clusters in the hierarchical cluster analysis: the mulched and fertilized, the nonmulched and fertilized, and the nonfertilized (Figure 3B), showing that both mulching and N fertilization affected the diazotroph community structure.

**FIGURE 4:**
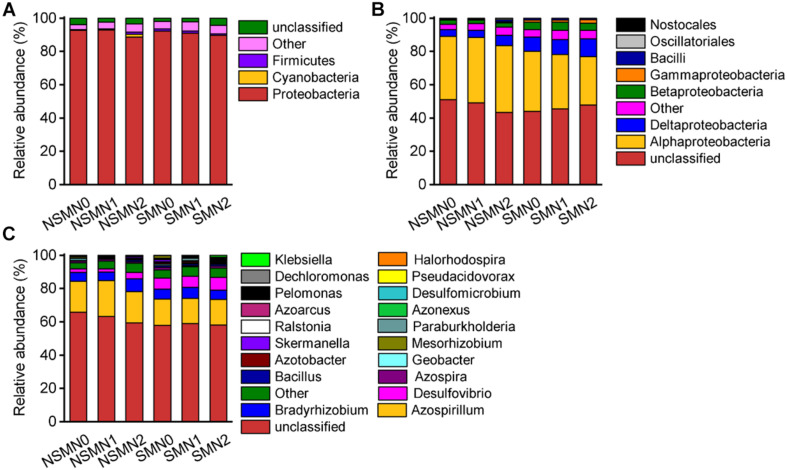
The community composition of diazotrophs in wheat rhizosphere soil under straw mulching and nitrogen fertilization treatments. **(A)** Phylum level; **(B)** class level; **(C)** genus level. NSM, no straw mulching; SM, straw mulching; N0, no nitrogen; N1, 120 kg N ha^− 1^; N2, 180 kg N ha^− 1^.

**FIGURE 5:**
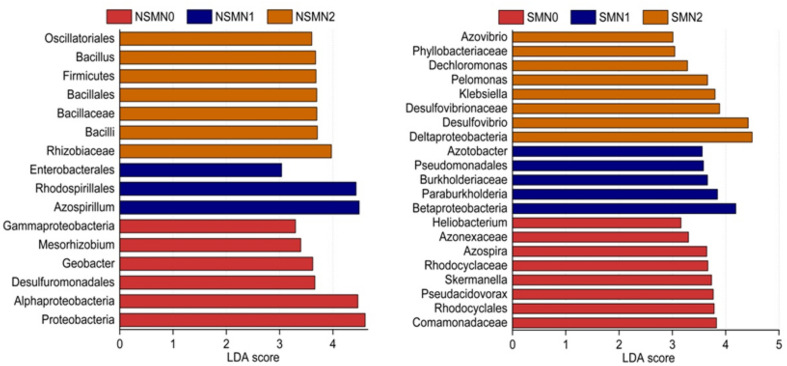
Differentially abundant diazotroph taxa in wheat rhizosphere soil under straw mulching and nitrogen fertilization treatments. Detected using linear discriminant analysis effect size analysis. NSM, no straw mulching; SM, straw mulching; N0, no nitrogen; N1, 120 kg N ha^− 1^; N2, 180 kg N ha^− 1^.

The relative abundance of the phylum *Proteobacteria* accounted for 88.5–92.9% of the total relative abundance in all the treatments (Figure 4A). The relative abundances of the classes *Deltaproteobacteria*, *Betaproteobacteria*, *Gammaproteobacteria*, and *Bacilli* and those of the genera *Azospirillum*, *Bradyrhizobium*, and *Desulfovibrio* were high in all treatments (Figures 4B,C). In the LEfSe analysis, 17 and 21 taxa in the nonmulched and the mulched treatments, respectively, had large effect sizes with LDA score >2.5 (Supplementary Table 2). At the genus level, the relative abundances of *Azospirillum, Bacillus*, and *Geobacter* were higher in the NSMs, and those of *Pseudacidovorax*, *Skermanella*, *Azospira*, *Paraburkholderia*, *Azotobacter*, *Desulfovibrio*, *Klebsiella*, and *Pelomonas* were higher in the mulched treatments (LDA score > 3.5) (Figure 5 and Supplementary Table 2).

### Correlation Between *nifH* Gene Abundance, Diazotrophic Community Diversity and Composition, and Soil Properties

**FIGURE 6:**
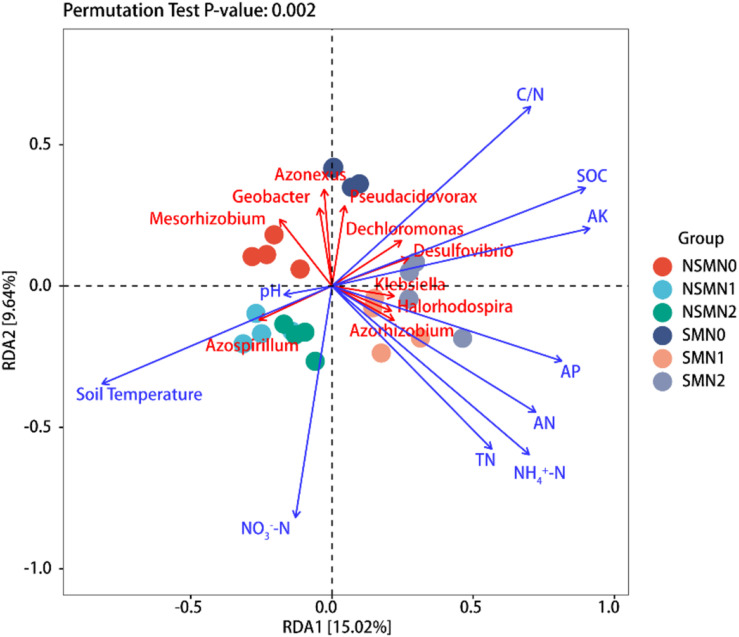
Redundancy analysis (RDA) of the *nifH* community and soil environmental factors in the wheat rhizosphere. SOC, soil organic carbon; TN, total nitrogen; AN, available nitrogen; NH_4_^+^-N, ammonium nitrogen; NO_3_^–^-N, nitrate nitrogen; AP, available phosphorus; AK, available potassium; NSM, no straw mulching; SM, straw mulching; N0, no nitrogen; N1, 120 kg N ha^− 1^; N2, 180 kg N ha^− 1^.

Shannon diversity correlated positively with SOC, TN, AN, NH_4_^+^-N, NO_3_^–^-N, AP, and AK contents and C:N ratio and negatively with soil temperature (*P* < 0.05), and the number of observed species correlated positively with SOC, AN, NO_3_^–^-N, AP, and AK contents and C:N ratio and negatively with soil temperature (*P* < 0.05) (Table 1). The abundance of *nifH* gene correlated positively with SOC, AK, AP, AN, NH_4_^+^-N, and NO_3_^–^-N contents and C:N ratio and negatively with soil temperature (*P* < 0.05) (Table 1). The relationships between soil physicochemical properties and the diazotroph community composition at the genus level were visualized using RDA (Figure 6 and Supplementary Table 3). Differences in community composition between the mulched and the NSMs were associated with differences in soil temperature, C:N ratio, and SOC and AK contents (Figure 6). The genus *Azospirillum* correlated positively with soil temperature, whereas the genera *Dechloromonas* and *Desulfovibrio* correlated positively with C:N ratio and SOC and AK contents. The differences between the fertilized and the nonfertilized treatments were associated with differences in NH_4_^+^-N, AN, AP, and TN contents. The genus *Azorhizobium* correlated positively and *Mesorhizobium* negatively with soil TN, NH4^+^-N, AN, and AP contents (Figure 6).

**TABLE 1:** The correlations of soil properties with α-diversity and *nifH* gene abundance in the wheat rhizosphere.

	pH	SOC	TN	C:N	AN	NH_4_^+^−N	NO_3_^−^−N	AP	AK	Soiltemperature
Shannon	–0.19	0.74**	0.55**	0.53**	0.65**	0.64**	0.73**	0.73**	0.73**	−0.64**
Observed species	0.02	0.63**	0.31	0.52**	0.44*	0.37	0.59**	0.59**	0.59**	−0.56**
*nifH* gene abundance	–0.16	0.65**	0.45	0.48*	0.56*	0.50*	0.66**	0.63**	0.66**	−0.56*

SOC, soil organic carbon; TN, total nitrogen; AN, available nitrogen; NH_4_^+^-N, ammonium nitrogen; NO_3_^–^-N, nitrate nitrogen; AP, available phosphorus; AK, available potassium; **and* indicate statistically significant correlation at *P* < 0.01 and *P* < 0.05, respectively.

## Discussion

Soil diazotrophs include Bacteria and Archaea that fix atmospheric nitrogen as free-living (e.g., *Azospirillum* and *Azotobacter*) or in symbiosis (e.g., *Bradyrhizobium* and *Rhizobium*) (De Bruijn, 2015). The abundance, diversity, and community composition of soil diazotrophs have been associated with various factors such as soil physicochemical characteristics and management strategies (Reardon et al., 2014). Fertilization and straw return change the environmental conditions and may affect the diazotrophs due to their individual responses to the changes in soil properties (Romero et al., 2012; Wang et al., 2016; Yang et al., 2019). We assessed the response of diazotrophs to straw mulching and N fertilization by quantifying and sequencing the *nifH* gene and noticed that the variation in diazotroph communities was more associated with straw mulching than with N fertilization. In previous studies, stubble retention and straw incorporation increased the abundance, diversity, and richness of the *nifH* carrying diazotrophs, which is possibly associated with the input of labile carbohydrates; monosaccharides and polysaccharides in straw provided an energy and nutrient source for the generally heterotrophic diazotrophs (Park et al., 2009). The N fertilization-accompanied soil acidification might in turn decrease the abundance and diversity of diazotrophs (Meng et al., 2012; Reardon et al., 2014). We found that the abundance and α-diversity of diazotrophs were greater the higher the fertilization level in the mulched treatments, whereas in the NSMs, richness was lowest with the highest N fertilization level, indicating that the slightly negative effect of N fertilization had been offset by mulching.

The availability of soil nutrients, which is highly responsive to straw and fertilizer input, is crucial for the establishment of soil diazotroph community structure (Reardon et al., 2014; Hu et al., 2019; Yang et al., 2019). In our study, Pearson correlation analysis showed that Shannon diversity was associated with differences in soil temperature, SOC, TN, AN, NH_4_^+^-N, NO_3_^–^-N, AP, and AK contents and C:N ratio, and the number of observed species was associated with differences in soil temperature; SOC, AN, NO_3_^–^-N, AP, and AK contents; and C:N ratio. In agreement, the results of RDA suggested that the differences in diazotroph communities were associated with differences in soil temperature; NH_4_^+^-N, AN, NO_3_^–^-N, AP, and TN contents; and C:N ratio. Especially, the differences between the mulched and NSMs possibly resulted from the higher SOC and AK contents in the mulched treatments.

Consistent with previous studies (Reardon et al., 2014; Lin et al., 2018; Hu et al., 2019, 2020), we observed that the relative abundances of Proteobacteria that play a vital role in carbon cycle and nitrogen mineralization (Wu et al., 2020) were high. However, it is worth noting that concluding absolute abundances directly from the relative abundance data is not possible, for example, due to bias introduced by the primers (McLaren et al., 2019). Consistent with Hu et al. (2019), the relative abundances of the class α-Proteobacteria and the genera *Azospirillum* within this class were high. *Azospirillum* is the best characterized genus of plant growth-promoting rhizobacteria and increased the yield and dry weight of wheat (Lin et al., 2018). The diazotroph communities may respond differentially to environmental factors and cultivation practices in different soil types (Wang C. et al., 2017; Lin et al., 2018). Contrary to Wang C. et al. (2017), in our study, *Azospirillum* correlated negatively with SOC content, possibly due to the differential response.

The soil N cycle is generally coupled with sulfur and iron cycles, and high N availability can increase the iron and sulfur cycle-related genes (Bao et al., 2014; Minamisawa et al., 2016). N limitation inhibited the growth of sulfate-reducing bacteria and the relative abundance of *Desulfovibrionales* that are mostly sulfate reducers increased with N fertilizer input (Sim et al., 2012; Li et al., 2018). In agreement, we found that the relative abundance of *Desulfovibrio* was the highest in the mulched and high N fertilization level treatment. In addition, straw mulching combined with N fertilization led to higher relative abundances of *Azotobacter*, *Klebsiella*, and *Paraburkholderia* that participate in the N cycle in soil ecosystems and have plant growth-promoting properties (Li et al., 2019; Patel et al., 2019; Castellano-Hinojosa and Strauss, 2020; Liu et al., 2020; Mukherjee et al., 2020; Ravi et al., 2020), suggesting that straw return may further contribute to the growth of plants.

In summary, our study demonstrated that straw mulching and N fertilization lead to changes in soil diazotroph community composition and to higher abundance, diversity, and richness of the *nifH* gene. The differences in community composition between the mulched and NSMs were associated with differences in soil temperature, C/N, SOC, and AK contents. Straw mulching combined with N fertilization was characterized by higher relative abundances of plant growth-promoting *Azotobacter*, *Klebsiella*, and *Paraburkholderia* genera. Overall, straw mulching and N fertilization were associated with changes in diazotroph community composition and increased the abundance of the *nifH* gene.

## Data Availability Statement

The nifH amplicon sequences were deposited in the National Center for Biotechnology Information (NCBI) GenBank database with the accession number SRP299232. The sequence of the nifH qPCR standard was deposited in the NCBI GenBank database with the accession number MW817080.

## Author Contributions

All authors listed have made substantial, direct and intellectual contribution to the work, and approved it for publication.

## Conflict of Interest

The authors declare that the research was conducted in the absence of any commercial or financial relationships that could be construed as a potential conflict of interest.
