# Supplementary material for: Straw Mulching and Nitrogen Fertilization Affect Diazotroph Communities in Wheat Rhizosphere
Source: Front Microbiol. 2021 May 21;12:658668. doi: 10.3389/fmicb.2021.658668 (PMC8175977; doi:10.3389/fmicb.2021.658668)
Supplement: Supplementary file 1 [file Data_Sheet_1.docx]

**Straw mulching and nitrogen fertilization affected diazotroph communities in wheat rhizosphere**

Songhe Chen^1†^, Xiaoling Xiang^1†^, Hongliang Ma^1^, Petri Penttinen^2^, Jiarong Zhao^1^, Han Li^1^, Rencai Gao^1^, Ting Zheng^1^, Gaoqiong Fan^1^[[1]](#footnote-1)^*^

*^1^Key Laboratory of Crop Eco-Physiology & farming system in Southwest China, Ministry of Agriculture,* *College of Agronomy*, *Sichuan Agricultural University,* *Chengdu, 611130, Sichuan Province, PR China;*

*^2^Department of Microbiology College of Resources, Sichuan Agricultural University, Chengdu, 611130, Sichuan Province, PR China.*

**SUPPLEMENTARY INFORMATION LIST**

**SUPPLEMENTARY TABLES**

**Supplementary Table S1**

**Supplementary Table S2**

**Supplementary Table S3**

**SUPPLEMENTARY TABLES**

**Table S1** PERMANOVA analysis of treatment effects on diazotroph community structure.

|  |  | p-value | Permutations |
| --- | --- | --- | --- |
| NSMN0 | NSMN1 | 0.025 | 999 |
|  | NSMN2 | 0.025 | 999 |
|  | SMN0 | 0.042 | 999 |
|  | SMN1 | 0.022 | 999 |
|  | SMN2 | 0.029 | 999 |
| NSMN1 | NSMN2 | 0.026 | 999 |
|  | SMN0 | 0.033 | 999 |
|  | SMN1 | 0.026 | 999 |
|  | SMN2 | 0.024 | 999 |
| NSMN2 | SMN0 | 0.031 | 999 |
|  | SMN1 | 0.031 | 999 |
|  | SMN2 | 0.035 | 999 |
| SMN0 | SMN1 | 0.033 | 999 |
|  | SMN2 | 0.026 | 999 |
| SMN1 | SMN2 | 0.049 | 999 |

**Table S2** Discriminant taxa significantly retrieved by LEfSe analysis for different treatments.

| Straw treatments | Nitrogen levels | LDA score | Taxonomy |
| --- | --- | --- | --- |
| NSM | N0 | 4.605 | *Proteobacteria* |
|  |  | 4.468 | *Alphaproteobacteria* |
|  |  | 3.661 | *Desulfuromonadales* |
|  |  | 3.621 | *Geobacter* |
|  |  | 3.397 | *Mesorhizobium* |
|  |  | 3.298 | *Gammaproteobacteria* |
|  | N1 | 4.493 | *Azospirillum* |
|  |  | 4.434 | *Rhodospirillales* |
|  |  | 3.038 | *Enterobacterales* |
|  |  | 3.972 | *Rhizobiaceae* |
|  | N2 | 3.708 | *Bacilli* |
|  |  | 3.698 | *Bacillaceae* |
|  |  | 3.697 | *Bacillales* |
|  |  | 3.683 | *Firmicutes* |
|  |  | 3.675 | *Bacillus* |
|  |  | 3.604 | *Oscillatoriales* |
|  |  | 3.596 | *Oscillatoriales* |
| SM | N0 | 3.828 | *Comamonadaceae* |
|  |  | 3.783 | *Rhodocyclales* |
|  |  | 3.766 | *Pseudacidovorax* |
|  |  | 3.737 | *Skermanella* |
|  |  | 3.667 | *Rhodocyclaceae* |
|  |  | 3.645 | *Azospira* |
|  |  | 3.303 | *Azonexaceae* |
|  |  | 3.161 | *Heliobacterium* |
|  | N1 | 4.191 | *Betaproteobacteria* |
|  |  | 3.847 | *Paraburkholderia* |
|  |  | 3.660 | *Burkholderiaceae* |
|  |  | 3.584 | *Pseudomonadales* |
|  |  | 3.561 | *Azotobacter* |
|  | N2 | 4.496 | *Deltaproteobacteria* |
|  |  | 4.422 | *Desulfovibrio* |
|  |  | 3.886 | *Desulfovibrionaceae* |
|  |  | 3.800 | *Klebsiella* |
|  |  | 3.660 | *Pelomonas* |
|  |  | 3.284 | *Dechloromonas* |
|  |  | 3.046 | *Phyllobacteriaceae* |
|  |  | 3.011 | *Azovibrio* |

NSM, no straw mulching; SM, straw mulching; N0, no nitrogen; N1, 120 kg N ha^-1^; N2, 180 kg N ha^-1^.

**Table S3 Physicochemical properties of wheat rhizosphere soil under straw mulching and nitrogen fertilization treatments.**

| Effects | pH | SOC (g/kg) | TN (g/kg) | C/N | AN (mg/kg) | NH_4_^+^-N (mg/kg) | NO_3_^-^-N (mg/kg) | AP (mg/kg) | AK (mg/kg) |
| --- | --- | --- | --- | --- | --- | --- | --- | --- | --- |
| SM | 8.02 | 13.5a | 1.02a | 13.25a | 65.1a | 3.89 | 8.60b | 9.02a | 201a |
| NSM | 8.04 | 9.49b | 0.97b | 9.80b | 61.6b | 3.05 | 11.20a | 8.06b | 170b |
| N0 | 8.02 | 11.4a | 0.90b | 12.49a | 59.4c | 2.35 | 6.08c | 7.98b | 181b |
| N1 | 8.02 | 11.3a | 1.04a | 10.92b | 63.9b | 3.95 | 10.9b | 8.80a | 188a |
| N2 | 8.05 | 11.8a | 1.05a | 11.16b | 66.8a | 4.11 | 12.8a | 8.85a | 186a |
| ANOVA | | | | | | | | | |
| M | 0.834 | 158.234^**^ | 65.131^**^ | 93.840** | 341.588^**^ | 91.898^**^ | 222.435^**^ | 23.899^*^ | 1688.099^**^ |
| N | 0.766 | 1.561 | 128.057^**^ | 12.120** | 50.316^**^ | 223.955^**^ | 280.951^**^ | 26.72^**^ | 6.748^*^ |
| M×N | 1.942 | 2.973 | 2.597 | 1.28 | 3.854 | 1.745 | 11.418^**^ | 1.134 | 1.002 |

NSM, no straw mulching; SM, straw mulching; N0, no nitrogen; N1, 120 kg N ha^-1^; N2, 180 kg N ha^-1^; M, mulching treatment; N, nitrogen fertilization treatment. SOC, Soil organic carbon; TN, Total nitrogen; AN, Available nitrogen; NH_4_^+^-N, Ammonium nitrogen; NO_3_^-^-N, Nitrate nitrogen; AP, Available phosphorus; AK, Available potassium. Different superscript letters in a column indicate statistically significant differences (*P* <0.05). ^*^, statistically significant difference (*P* < 0.05); ^**^, statistically significant difference (*P* < 0.01).

1. *Corresponding author: fangao20056@126.com (G. Fan.)

   † These authors contributed equally to this work. [↑](#footnote-ref-1)
